# Multilocus Variable-Number Tandem-Repeat Analysis, Pulsed-Field Gel Electrophoresis, and Antimicrobial Susceptibility Patterns in Discrimination of Sporadic and Outbreak-Related Strains of *Yersinia enterocolitica*

**DOI:** 10.1186/1471-2180-11-42

**Published:** 2011-02-25

**Authors:** Leila M Sihvonen, Susanna Toivonen, Kaisa Haukka, Markku Kuusi, Mikael Skurnik, Anja Siitonen

**Affiliations:** 1 Department of Infectious Disease Surveillance and Control, Bacteriology Unit, National Institute for Health and Welfare (THL), Helsinki, FI-00271, Finland; 2 Department of Infectious Disease Surveillance and Control, Epidemiological Surveillance and Response Unit, National Institute for Health and Welfare (THL), Helsinki, FI-00271, Finland; 3 Department of Bacteriology and Immunology, Haartman Institute, University of Helsinki, FI-00014, Finland; 4 Helsinki University Central Hospital Laboratory Diagnostics, Helsinki, Finland

## Abstract

**Background:**

We assessed the potential of multilocus variable-number tandem-repeat analysis (MLVA), pulsed-field gel electrophoresis (PFGE), and antimicrobial susceptibility testing for discriminating 104 sporadic and outbreak-related *Yersinia enterocolitica *(YE) bio/serotype 3-4/O:3 and 2/O:9 isolates. MLVA using six VNTR markers was performed in two separate multiplex PCRs, and the fluorescently labeled PCR products were accurately sized on an automated DNA sequencer.

**Results:**

MLVA discriminated 82 sporadic YE 3-4/O:3 and 2/O:9 strains into 77 types, whereas PFGE with the restriction enzyme *Not*I discriminated the strains into 23 different PFGE pulsotypes. The discriminatory index for a sporadic strain was 0.862 for PFGE and 0.999 for MLVA. MLVA confirmed that a foodborne outbreak in the city of Kotka, Finland in 2003 had been caused by a multiresistant YE 4/O:3 strain that was distinctly different from those of epidemiologically unrelated strains with an identical PFGE pulsotype. The multiresistance of *Y. enterocolitica *strains (19% of the sporadic strains) correlated significantly (p = 0.002) with travel abroad. All of the multiresistant *Y. enterocolitica *strains belonged to four PFGE pulsotypes that did not contain any susceptible strains. Resistance to nalidixic acid was related to changes in codons 83 or 87 that stemmed from mutations in the *gyrA *gene. The conjugation experiments demonstrated that resistance to CHL, STR, and SUL was carried by a conjugative plasmid.

**Conclusions:**

MLVA using six loci had better discriminatory power than PFGE with the *Not*I enzyme. MLVA was also a less labor-intensive method than PFGE and the results were easier to analyze. The conjugation experiments demonstrated that a resistance plasmid can easily be transferred between *Y. enterocolitica *strains. Antimicrobial multiresistance of *Y. enterocolitica *strains was significantly associated with travel abroad.

## Background

*Yersinia enterocolitica *(YE) is an enteropathogenic bacterium transmitted via food or water and may cause sporadic infections as well as foodborne outbreaks of yersiniosis [1-5]. The symptoms of yersiniosis range from mild diarrhea to severe clinical manifestations and post-infectious complications such as reactive arthritis, myocarditis, glomerulonephritis, and erythema nodosum [6]. *Y. enterocolitica *can be divided into six biotypes, of which biotypes 1B and 2-5 are known to be pathogenic to humans.

At present, pulsed-field gel electrophoresis (PFGE) is commonly used to discriminate between *Y. enterocolitica *strains. However, there are no standard PFGE procedures or databases similar to those, *e.g.*, for *Escherichia coli *O157:H7, *Salmonella*, and *Shigella *standardized by PulseNet [7]. Most of the restriction enzymes used in PFGE for *Y. enterocolitica *produce patterns with a high number of bands that are not ideal for analysis. Furthermore, the global homogeneity of the pulsotypes among *Y. enterocolitica *4/O:3 is high and different pulsotypes often display only minor differences [8-11]. However, the discriminatory power of PFGE has been improved by using more than one restriction enzyme [12].

Most bacterial genomes contain repeats of DNA sequences called "variable-number tandem repeats" (VNTR). These VNTR regions can be applied in the PCR-based subtyping of strains by multilocus variable-number tandem-repeat analysis (MLVA). MLVA is increasingly used for typing, surveillance and epidemiological investigations of pathogenic bacteria [13]. A study investigating the development of an MLVA subtyping method to be used for *Y. enterocolitica *4/O:3, based on six loci, was reported recently [14].

Although yersiniosis is seldom treated with antimicrobials, medication may be required, for example in the case of immuno-compromised patients. *Y. enterocolitica *is a known ß-lactamase producer and thus is resistant to ß-lactam antibiotics such as ampicillin, carbenicillin, penicillin, and first-generation cephalosporins [15-20]. In recent studies done in Switzerland, the USA, Germany, and Austria, *Y. enterocolitica *strains have shown high susceptibility to antimicrobials other than ß-lactams [21-24]. However, multiresistant *Y. enterocolitica *strains have also been reported, *e.g.*, from Spain and Brazil [16,25,26]. The antimicrobial resistance of *Y. enterocolitica *has not been monitored regularly in Finland although the surveillance of antimicrobial resistance would be useful for epidemiological studies. Over 20 years ago, 186 Finnish *Y. enterocolitica *strains were studied and found to be resistant only to ampicillin and susceptible to ceftriaxone, tetracycline, sulpha-trimethoprim, and ciprofloxacin [27].

The aim of the present study was to determine how MLVA using fluorescently labeled primers and fragment analysis compares to PFGE in its discriminatory power with regard to the sporadic and outbreak-related strains of YE bio/serotypes 4/O:3. We included traditional antimicrobial susceptibility testing in our study to see whether it provides additional information for the genotypic analysis concerning, *e.g.*, the geographical source of infection. We therefore used MLVA and PFGE to type 104 sporadic and outbreak-associated *Y. enterocolitica *strains and determined the sensitivity of the strains to 12 antimicrobial agents. In addition, we studied the genetic basis of the antimicrobial resistance that was detected.

## Results

### Sporadic strains

**Figure 1:**
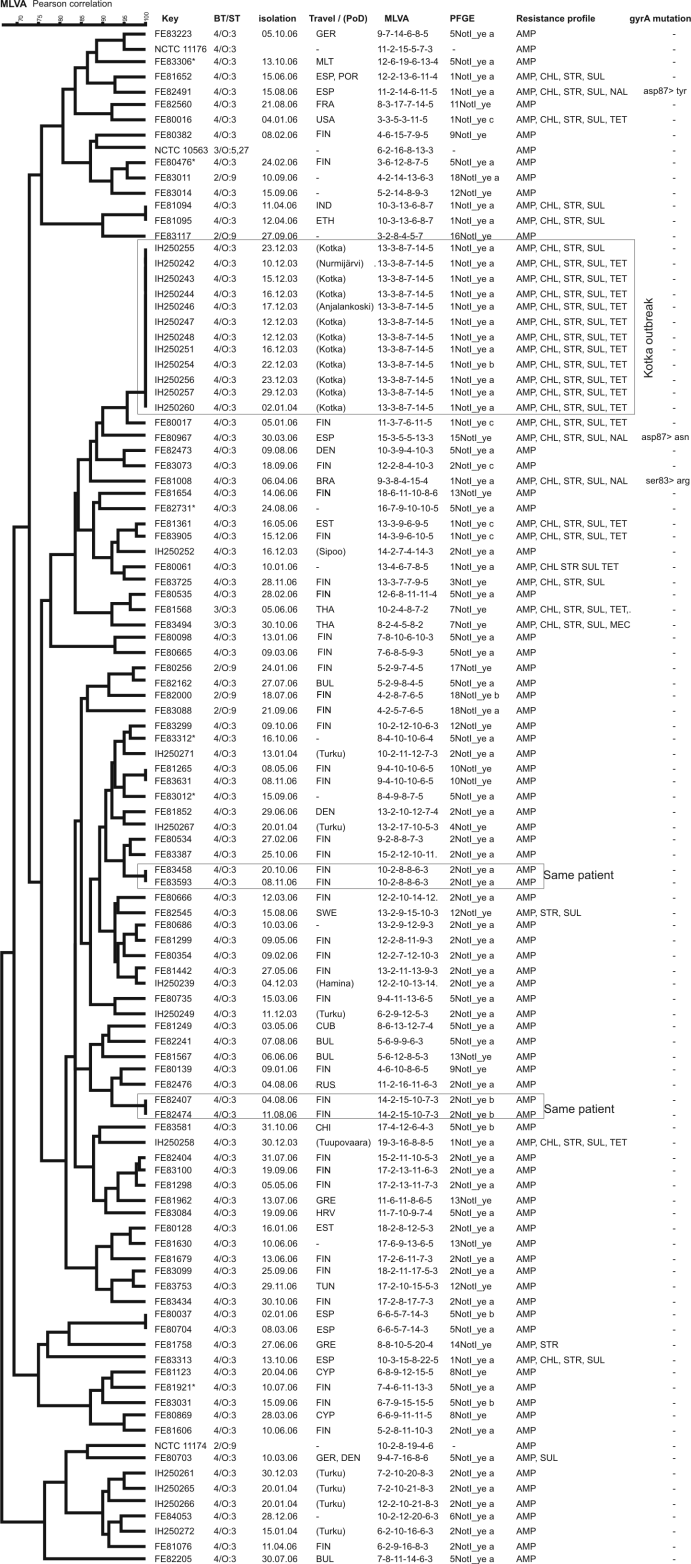
**MLVA tree**. UPGMA clustering of the MLVA results, with Pearson's correlation similarity coefficients, was performed using Bionumerics version 5.10. The key column provides the strain ID. Information on bio/serotype, travel abroad or place of domicile (PoD), MLVA types named as a string of six numbers showing the actual number of repeat units in each of the six loci, PFGE pulsotype, and antimicrobial resistance are presented in the columns. *Strains isolated from a 1-year old children in the case of a suspected outbreak with PFGE pulsotype 5NotI_ye_a.

**Figure 2:**
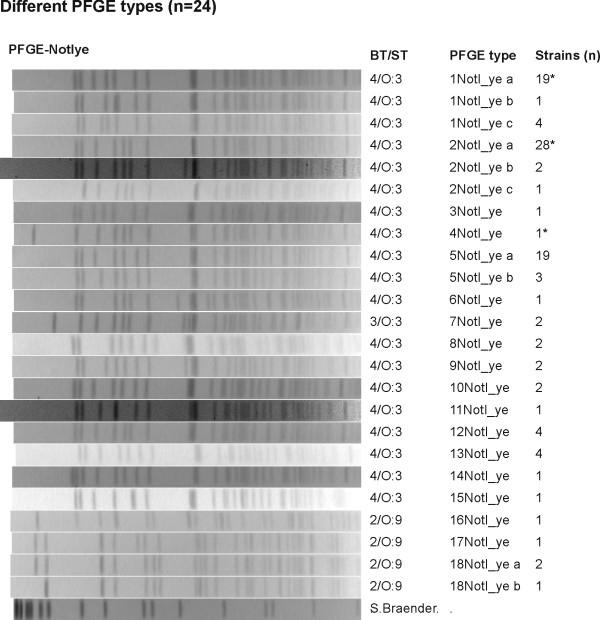
**PFGE types of the studied strains**. All 24 representative PFGE types of 104 strains in the present study. * The strain number includes the outbreak types.

The strains isolated in 2006 (n = 82) were discriminated into 77 types by MLVA (Figure 1) and into 23 pulsotypes by PFGE (Figure 2). There were two YE 4/O:3 strains with identical MLVA types in only five cases. In two of these cases, the identical strains had been isolated from one patient 7 days apart and from another patient 19 days apart. The discriminatory index for sporadic strains was 0.862 for PFGE and 0.999 for MLVA.

The six loci used in MLVA V2A exhibited the highest discriminatory power (DI = 92%), resolving 17 different alleles. The least variation was observed for locus V9 (DI = 62%), which yielded only six different alleles, *i.e.*, 2-7 repeats of a repetitive sequence 12 bp in length. The discriminatory indexes of loci V4, V5, V6, and V7 were 71, 89, 91, and 90%, respectively. The fragment sizes defined by the capillary electrophoresis of the six VNTR loci and the number of repeats confirmed by DNA sequencing are shown in Table 1.

**Table 1:** Diversity of VNTR alleles.

Number of the repeats	V2A TCTCAC (bp)	n†	V4 CGGCAAC (bp)	n	V5 GGTGCA (bp)	n	V6 GACTCA (bp)	n	V7 GTGCTG (bp)	n	V9 ATGTCGGTAGAA (bp)	n
2	-		119*	49			-		-		108	2
3	246*	2	126*	26			182	1	-		120*	52
4	252	5	133*	9	199	2	188*	5	195*	4	132	8
5	258	6	140	0	205	4	194	5	201*	8	144*	40
6	264	10	147	15	211	3	200*	11	207	19	156	2
7	270	6	154*	4	217	6	206*	21	213	13	168*	3
8	276	6	161	4	223*	25	212*	13	219	12	-	
9	282*	7	-		229	17	218	2	225	9	-	
10	288	10	-		235	15	224	12	231	9	-	
11	294	6	-		241	8	230	9	237	7	-	
12	300	10	-		247	6	236	10	243	1	-	
13	306	20	-		253	6	242	4	249	4	-	
14	312	4	-		259	5	248	2	255*	16	-	
15	318	3	-		265*	5	254	3	261	3	-	
16	324	1	-		271	3	260	3	267	-	-	
17	330*	7	-		277	1	266	2	273	-	-	
18	336	3	-		283	-	272	-	279	-	-	
19	342	1	-		290	1	278	-	285	-	-	
20	-		-		-		284	2	291	1	-	
21	-		-		-		300	2	297	-	-	
22	-		-		-				303*	1	-	

Fragment sizes (bp) defined by capillary electrophoresis of VNTR alleles with different number of repeats and their diversity in 107 studied *Y. enterocolitica *strains.

* Alleles were sequenced to confirm the number of repeats.

**† **n = number of strains

### Suspected outbreak strains

The suspected outbreak strains (n = 22) from December 2003 to January 2004 represented four PFGE pulsotypes, which were further typed into 11 MLVA types. Twelve of the strains were identical in MLVA type. Eleven of these strains with identical MLVA types were isolated from the patients with an epidemiological connection to the disease outbreak. The 12 strains with identical MLVA type represented 2 slightly different (only one band difference) PFGE pulsotypes (Figure 2) and were multiresistant to antimicrobials (Figure 1). Among these strains, eleven were resistant to AMP, CHL, STR SUL, and TET; one strain was susceptible to TET. The suspected outbreak strains with different MLVA types did not have a proved connection to the city of Kotka, Finland. Nine of these strains were susceptible to all the tested antimicrobials except AMP and eight of them shared the same PFGE type. One of the strains (IH250258) had an antimicrobial resistance profile and a PFGE pulsotype identical to those of the outbreak strains. However, the different MLVA type and the lack of epidemiological connection distinguished this particular case from the outbreak-associated cases (Figure 2). Suspected YE 4/O:3 outbreak strains isolated in 2006 from six 1-year-old children displayed the same PFGE pulsotype (5NotI_ye a). However, the MLVA discriminated all six strains.

### Association between the antimicrobial resistance and travel

All the *Y. enterocolitica *strains studied here were resistant to ampicillin. Fifteen (19%) of 80 sporadic strains isolated in 2006 from 80 patients were resistant to four or five of the antimicrobials tested (Table 2). The multiresistant strains belonged to certain PFGE pulsotypes (1NotI_ye, 3NotI_ye, 7NotI_ye, 15NotI_ye) that did not contain any susceptible strains. The travel history of 70 of the 80 patients was known. Of these patients, 46% (32/70) had traveled abroad before the onset of symptoms. Travel abroad was significantly (p = 0.002) associated with the antimicrobial multiresistance of *Y. enterocolitica*: 34% (11/32) of the patients with and 5% (2/38) of the patients without a trip abroad had a multiresistant *Y. enterocolitica *strain. Three strains resistant to nalidixic acid had decreased susceptibility (0.25, 0.25, or 0.5 mg/L) to ciprofloxacin in MIC determination. Sequencing of these three nalidixic acid resistant strains revealed amino acid changes due to the point mutations in the *gyrA *gene; *i.e.*, Ser83 to Arg or Asp87 to Asn or Asp87 to Tyr.

**Table 2:** Antimicrobial resistance and travelling.

Resistance profile	Suspected outbreak YE 4/O:3 (n = 22)	Sporadic YE 4/O:3 (n = 75)	Sporadic YE 3/O:3 (n = 2)	Sporadic YE 2/O:9 (n = 5)
AMP CHL STR SUL NAL	-	3 (100%)*	-	-
AMP CHL STR SUL TET	12 (0%)	5 (40%)	-	-
AMP CHL STR SUL TET MEC	-	-	1 (100%)	-
AMP CHL STR SUL	1 (0%)	5 (80%)	-	-
AMP CHL STR SUL MEC	-	-	1 (100%)	-
AMP SUL	-	1 (100%)	-	-
AMP STR	-	1	-	-
AMP MEC	-	-	-	-
AMP	9 (0%)	61 (28%)	-	5 (0%)

Antimicrobial resistance profiles of the sporadic *Y. enterocolitica *strains isolated in Finland in 2006 and suspected outbreak strains from 2003-2004 and related travel information.

* The percentage of the patients who had reported having traveled abroad before getting ill is in the parenthesis.

### Conjugation of resistance plasmid

In the conjugation experiment, a sporadic YE 4/O:3 strain FE81008 (resistant to AMP, CHL, STR, SUL, and NAL) was able to transfer the CHL, STR, and SUL resistances to strain YeO3-U by conjugation. The conjugation frequency was 10^-5^-10^-6^. This indicated that the genes encoding resistance to CHL, STR, and SUL were carried on a conjugative plasmid. Indeed, plasmid isolation demonstrated that the recipient strain had received a large 30-40 kb plasmid.

## Discussion

In our study, MLVA typing using fluorescently labeled primers and fragment analysis was shown to be a high-resolution discriminatory method for epidemiological investigations of *Y. enterocolitica*. In the present study, the discriminatory power of MLVA was 99.9% while that of *Not*I PFGE was 87.9%. Our results were in agreement to those obtained by Gierczyński and colleagues [14] who demonstrated that the used MLVA markers are highly discriminatory and added the evidence that this method can successfully be applied for the outbreak strains of *Y. enterocolitica *ssp. *palearctica *biotypes 2 and 4. In the present study, only the VNTR loci V2A, V4 and V5 were found in six BT 1A strains tested with the MLVA method (data not shown). Another MLVA method designed using *Y. enterocolitica *ssp. *enterocolitica *strain 8081 whole genome and with four loci was introduced recently [28]. The method showed potential for the epidemiological investigation for YE biotype 1A strains with DI of 87% and worked also for six tested BT 2 and BT4 strains [28]. The discriminatory power of PFGE can be improved by using more than one restriction enzyme. For instance, the discriminatory index of 74% achieved with *Not*I PFGE was increased to 93% by using further characterization with *Apa*I and *Xho*I enzymes of 128 YE 4/O:3 strains [29]. However, both the time required and the costs of PFGE rise considerably when several restriction enzymes are used. The amount of working time needed for the PFGE protocol with one enzyme is two to three days, MLVA using fragment analysis can be done in one day.

In December 2003, authorities from the city of Kotka, Finland reported an outbreak of gastroenteritis. Investigations revealed that it was caused by *Y. enterocolitica *4/O:3 [30]. Approximately 30 people fell ill; 12 patients had culture-confirmed, multiresistant YE 4/O:3 infection. Three of them had appendectomies before the disease was recognized as yersiniosis. Most of the patients had abdominal pain (94%), fever (78%), and diarrhea (72%). Most of the patients had eaten in the same cafeteria in the Port of Kotka between November 25 and December 15, 2003. However, the contaminated food source was not discovered, as the food served in that cafeteria was no longer available for microbiological investigations. In addition, a cohort study among cafeteria users did not show a significant association between any food and illness. During a microbiological sampling of the cafeteria's kitchen a month later, in January 2004, hygienists noticed some shortcomings in food handling and hygiene practices that increased the possibility of cross-contamination in the cafeteria. While no YE 4/O:3 strains were found in the specimens collected from the cafeteria, YE biotype 1A strains were isolated from iceberg lettuce imported from Spain and from domestic carrots. Unfortunately, the antimicrobial susceptibilities of these strains are not known. At the time of the outbreak in Kotka, there were around 20 confirmed YE 4/O:3 cases in other locations in Finland, mainly in the Turku area. The cases were suspected to be linked with the larger outbreak, but no epidemiological evidence for this was found.

MLVA played a key role in confirming that the cases which occurred in the city of Kotka in 2003 belonged to a single outbreak: 12 isolates representing the Kotka outbreak were clonal by MLVA, and differed distinctly from those of epidemiologically unrelated strains that shared the same PFGE pulsotype. Another suspicion of outbreak was refuted by MLVA: six 1-year-old children had been infected in 2006 by YE 4/O:3 strains that shared the same PFGE pulsotype (5NotI_ye a). Interviews, however, revealed no epidemiological connection between the cases. All of these strains which shared the same PFGE pulsotype were found to be of different types in MLVA. We also detected some evidence that the MLVA method can be as useful with YE 2/O:9 outbreaks as it was with YE 4/O:3. In a household outbreak in 2009, a mother and two children had YE 2/O:9 strains found to be identical in MLVA (data not shown here). MLVA also identified identical YE 2/O:9 strains in a school/day care center outbreak that occurred in Finland in 2010 (data not shown here).

Support was obtained for genetic stability among sporadic cases, since two MLVA-typed strains were isolated twice from the same patient at intervals of 7 or 19 days. In both cases, the MLVA and PFGE types remained identical. Similar observations of the stability of the *Y. enterocolitica *MLVA markers' loci *in vivo *had also been reported earlier [14]. Genetic events will eventually alter the MLVA patterns, but the rate of alteration is not known. However, previous studies confirmed that the MLVA type remained the same after as many as 20 serial passages of colony plating [14].

Our previous case-control study revealed that travel abroad was a risk factor for *Y. enterocolitica *infection in Finland [31]. In the present study, we found a statistically significant association between the antimicrobial multiresistance of YE strains and travel. The results indicate that a considerable number of multiresistant *Y. enterocolitica *infections are actually imported, and that domestic *Y. enterocolitica *strains in Finland are rather susceptible to antimicrobials. For instance, all of the nalidixic acid-resistant strains were isolated from patients who had been infected while on vacation in Spain or Brazil, countries where multiresistant *Y. enterocolitica *strains have been described previously [16,25,26]. The multiresistant strains belonged to certain PFGE pulsotypes, which were not found among susceptible strains. This is perhaps due to the DNA of the resistance plasmid. The MLVA types were so varied that no hint of the origin of the strains could be obtained on that basis.

In the outbreak that occurred in Kotka, the patients had not been abroad before falling ill. However, the antimicrobial multiresistance of the outbreak strain nevertheless suggests that the strain originated from abroad. Spanish iceberg lettuce, at least, had been used in the cafeteria. In 2005 *Salmonella enterica *serotype Typhimurium, with a resistance profile identical to that detected now for the *Y. enterocolitica *outbreak strain, was isolated in an outbreak situation in Finland and traced to iceberg lettuce imported from Spain [32].

The resistance of *Y. enterocolitica *to NAL is based on point mutations in the fluoroquinolone resistance-determining regions of *gyrA *[26,33]. In our study, the strains resistant to NAL had amino acid changes stemming from point mutations in the *gyrA *gene: *i.e.*, either Ser83Arg, Asp87Tyr, or Asp87Asn. Two of these mutations are identical to those reported previously for fluoroquinolone-resistant *Y. enterocolitica *strains [33].

Conjugation experiments confirmed that in *Y. enterocolitica*, the antibiotic resistance to CHL, STR, and SUL, at least, is encoded on a large conjugative plasmid and can easily be transferred to a susceptible *Y. enterocolitica *strain. Conjugative plasmids that carry antibiotic resistance genes have been isolated from a variety of clinical strains, but reports of this for *Y. enterocolitica *are rare. Hundreds of different antibiotic resistance cassettes have been identified as residing on mobile resistance integrons [34]; owing to the cassette nature of the resistance genes, they can easily change the resistance repertoire. In fact, one of the outbreak strains in our study had altered antimicrobial resistance and lacked resistance to TET. A study on the persistence of TET-resistant *E. coli *in colonic microbiota observed that three out of 13 strains lost TET resistance during intestinal colonization [35].

## Conclusions

MLVA was less labor-intensive than PFGE and the results were easier to analyze, especially because they were independent of subjective interpretation. PFGE can still be useful for surveillance of the sources and transmission routes of sporadic *Y. enterocolitica *strains in future. However, for outbreak investigations, MLVA offers a powerful tool for the discrimination of *Y. enterocolitica *strains. More sporadic and outbreak *Y. enterocolitica *strains should be subjected to MLVA typing in order to determine whether this method could be considered for use as a new gold standard for outbreak investigations of *Y. enterocolitica*. This study revealed that multiresistant *Y. enterocolitica *strains do appear in Finland, but that the multiresistance was mainly associated with travel. All three nalidixic acid resistant strains were associated with travel to Spain or Brazil. Interestingly, all outbreak strains studied here were also multiresistant. Thus, traditional susceptibility testing provides additional information useful for genetic typing methods in epidemiological investigations.

## Methods

### Bacterial strains

Sporadic *Y. enterocolitica *strains (n = 82) of bio/serotype 4/O:3 (n = 75), 3/O:3 (n = 2), 2/O:9 (n = 5) isolated in 2006 from fecal samples of 80 Finnish patients in ten regional clinical microbiology laboratories were used in the study. The patients' mean age was 34 years (range 0.6-80); 55% of them were men. Isolation and identification of the strains were described previously [36]. In addition, 22 clinical *Y. enterocolitica *strains isolated between December 2003 and January 2004, and suspected of being associated with a *Y. enterocolitica *outbreak in Kotka, were studied.

### MLVA

For MLVA, we had three additional reference strains: NCTC 1176 (4/O:3); NCTC 11174 (2/O:9); and NCTC 10563 (3/O:5,27). DNA was extracted from the strains using the Jet Flex Extraction Kit (Genomed; Löhne, Germany) according to the instructions provided by the manufacturer and eluated in 100 μL TE-buffer. In the MLVA analysis, six known VNTR loci of the strains were amplified in two multiplex PCRs. Previously described primers [14] were labeled with ABI PRISM^® ^fluorescent dyes, PET, NED, 6-FAM, or VIC (Applied Biosystems, Foster City, CA). Primers were used in two separate multiplex PCRs with the VNTR loci of V2A (PET), V4 (NED), and V6 (6-FAM), as well as V5 (NED), V7 (VIC), and V9 (PET). Multiplex PCRs were performed with QIAGEN Multiplex PCR kit (Qiagen, Hilden, Germany) according to the manufacturer's instructions in a total volume of 25 μl. The primer concentrations were 0.2 μM (V2A), 0.16 μM (V4), and 0.2 μM (V6) in the first PCR and 0.2 μM (V5), 0.2 μM (V7), 0.12 μM (V9) in the second PCR. The template DNA concentration was approx. 10 ng. Touchdown PCR was performed with 15 min initial denaturation at 95°C, followed by 9 cycles 30 s denaturation at 95°C, 30 s annealing at 63°C-55°C (decreasing by 1°C with every cycle), and elongation at 72°C with an additional 25 cycles with annealing 30 s at 58°C.

The two PCR products of each strain were mixed, diluted to 1/500 in sterile water, and run in capillary electrophoresis with an ABI 3730xl DNA Analyzer (Applied Biosystems, Foster City, CA) using G5 (DS-33) fragment analysis chemistry according to the manufacturer's instructions. The GeneScan™ 600 LIZ^® ^(Applied Biosystems) was used as an internal size standard and the data were analyzed using GeneMapper v4.0 software (Applied Biosystems). MLVA types were named as a string of six numbers showing the actual number of repeat units in each of the six loci.

### DNA sequencing

The selected repeats (Table 1) were sequenced in both directions with MLVA primers [14]. The *gyrA *gene PCR was performed for 77 sporadic *Y. enterocolitica *strains of bio/serotypes 4/O:3 and 3/O:3 with primers gyrAY1 (5'-CGC GTA CTG TTT GCG ATG AA-3') and gyrAY2 (5'-CGG AGT CAC CAT CGA CGG AA-3') as earlier described (35) (GenBank/EMBL/DDBJ accession numbers FN821873-FN821949). Sequencing was done in both directions with a Big Dye Terminator v1.1 Cycle Sequencing Kit (Applied Biosystems) with an ABI 3730xl DNA Analyzer (Applied Biosystems).

### PGFE

PFGE was performed using the previously described protocol for *Salmonella *[7,37] with modifications: Strains were cultured overnight at 30°C on R1-agar and suspended in CBS-buffer (100 mM Tris:100 MM EDTA, pH 8.0) to a final turbidity of 0.38-0.39 at A_480_. Lysozyme (Roche Diagnostics GmbH, Mannheim, Germany) was added to the 400 μl bacterial suspensions to reach a final concentration of 1 mg/ml. The tubes were mixed and incubated for 15 min at 37°C and then heated to 50°C, after which 400 μl of 1% agarose (SeaKem Gold Agarose, Cambrex Bio Science Rockland, Inc, USA) and proteinase K (at a final concentration of 0.24 mg/ml, Roche Diagnostics GmbH, Mannheim, Germany) were added. The tube contents were cast into plugs, which were transferred into 3 ml of lysis buffer (50 mM Tris:50 mM EDTA, pH8.0 + 1% Sarcosyl) containing 1 mg/ml of proteinase K. The plugs were incubated at 54°C for 2 h and rinsed three times in sterile water and three times in TE buffer at 50°C. The plugs were then stored in 1 × TE buffer at 4°C. The released genomic DNA in the plugs was digested overnight at 37°C with 8 U of the restriction enzyme *Not*I (New England Biolabs, Ipswich, MA, USA). Electrophoresis was carried out in a 1% agarose gel in 0.5 × TBE buffer at 14°C with a switching time of 1 to 18 s for 40 h at 14°C with CHEF Mapper system (Bio-Rad Laboratories, Richmond, California). DNA of the *Salmonella enterica *serotype Braenderup strain H9812, digested with *Xba*I (Roche GmbH, Mannheim, Germany), was used as a size marker. The PFGE types were analyzed with Bionumerics v. 5.10 software (Applied Maths, Sint-Martens-Latem, Belgium). DNA bands smaller than 54.7 kb were excluded from the analysis.

### Discriminatory index of PFGE and MLVA

Simpson's Index of diversity was used to calculate the discriminatory index (DI) of PFGE and MLVA [38]. In addition, the DIs of each MLVA locus was calculated.

### Susceptibility testing

The antimicrobial susceptibility of the *Y. enterocolitica *isolates was determined using a set of 12 antimicrobials: ampicillin (AMP); chloramphenicol (CHL); streptomycin (STR); gentamicin (GEN); sulfonamide (SUL); tetracycline (TET); trimethoprim (TMP); ciprofloxacin (CIP); nalidixic acid (NAL); cefotaxime (CEF); mecillinam (MEC); and imipenem (IMI). The susceptibility tests were done using the agar diffusion technique on Mueller-Hinton agar according to the CLSI guidelines [39]. A strain resistant to at least four antimicrobials was called multiresistant. The minimal inhibitory concentration (MIC) for ciprofloxacin (CIP) was determined by the E-test (AB Biodisk, Solna, Sweden) for the isolates resistant to nalidixic acid, following the recommended MIC breakpoints S ≤1 mg/L and R ≥4 mg/L [39]. MIC 0.125-1.0 mg/L was considered to indicate reduced susceptibility to ciprofloxacin [40].

### Conjugation experiments

In conjugation experiments, the multiresistant (AMP, CHL, STR, SUL, NAL) strain YE 4/O:3 FE81008 was used as a donor strain and the kanamycin (KAN) resistant strain YeO3-U [41] as a recipient strain. Briefly, the donor strain and the recipient strain were grown overnight at room temperature shaking in 5 ml of Luria broth (LB). The cultures were refreshed by diluting them 1:10 in LB and grown for 2-3 h to get them into the exponential phase. The donor strain was grown in static culture. The bacteria were then pelleted by centrifugation and resuspended in 1 ml of PBS. After the OD600 were determined, the suspensions were mixed 1:1 and small droplets of the mixture were pipetted onto a Luria-agar plate and incubated overnight at room temperature. Only the donor or the recipient bacteria was pipetted onto the control plates. The plates were incubated overnight after which the bacteria were collected from the plates into ca. 1 ml of PBS. Several dilutions were spread on selective plates containing CHL, KAN, or both CHL and KAN. The conjugation frequency was calculated on the basis of the proportion of CHL KAN double-resistant colonies among the CHL-resistant colonies. The resistance of the CHL KAN double-resistant colonies to the other antimicrobials was tested as described above. Plasmid isolation from 100 ml cultures of the strains was performed using the E.Z.N.A plasmid midiprep kit (Omega Bio-Tek Inc., Norcross, GA, USA) according to the protocol provided by the manufacturer, and the plasmids were detected by running in a 1% w/v agarose gel.

### Travel information and statistical method

Data on the patients' travel abroad were collected from the National Infectious Disease Register and from the notes of the laboratories sending the *Yersinia *strains for further typing. The association between travel and multiresistance was analyzed by using the chi-square method with the EpiInfo™ version 3.4.3. A p-value below 0.05 was considered to indicate statistical significance.

The study was approved by the Ethics Committee of National Institute for Health and Welfare, THL. For this study informed consents were not required as only the isolated bacterial strains of the fecal samples were studied and not the individuals themselves.

## Authors' contributions

LMS participated in the design of the study, did or supervised the MLVA, PFGE, DNA sequencing, and antimicrobial susceptibility testing, carried out the data analysis, and drafted the manuscript. ST performed the conjugation experiment. KH participated in the design of the study and drafting of the manuscript. MK did the epidemiological investigations of the study and edited the manuscript. MS designed the conjugation experiment and participated in drafting of the manuscript. AS obtained the funding, conceived the study, and edited the manuscript. All of the authors have read and approved the final manuscript.
